# Improvement in fast-track hip and knee arthroplasty: a prospective multicentre study of 36,935 procedures from 2010 to 2017

**DOI:** 10.1038/s41598-020-77127-6

**Published:** 2020-12-04

**Authors:** Pelle Baggesgaard Petersen, Henrik Kehlet, Christoffer Calov Jørgensen, The Lundbeck Foundation Centre for Fast-track Hip and Knee Replacement Collaborative Group, Frank Madsen, Torben Bæk Hansen, Kirill Gromov, Mogens Laursen, Lars Tambour Hansen, Per Kjærsgaard-Andersen, Soren Solgaard, Niels Harry Krarup, Jens Bagger

**Affiliations:** 1 Section for Surgical Pathophysiology, 7621, Copenhagen University Hospital, Rigshospitalet, Blegdamsvej 9, 2100 Copenhagen, Denmark; 2 Lundbeck Foundation Centre for Fast-Track Hip and Knee Arthroplasty, Copenhagen, Denmark; 3 Department of Orthopedics, Aarhus University Hospital, Aarhus, Denmark; 4 Department of Orthopedics, Regional Hospital Holstebro and University of Aarhus, Holstebro, Denmark; 5 Department of Orthopedics, Hvidovre Hospital, Hvidovre, Denmark; 6 Aalborg University Hospital Northern Orthopaedic Division, Aalborg, Denmark; 7 Department of Orthopedics, Sydvestjysk Hospital Esbjerg/Grindsted, Grindsted, Denmark; 8 Department of Orthopedics, Vejle Hospital, Vejle, Denmark; 9 Department of Orthopedics, Gentofte University Hospital, Copenhagen, Denmark; 10 Department of Orthopedics, Viborg Hospital, Viborg, Denmark; 11 Department of Orthopaedic Surgery, Copenhagen University Hospital Bispebjerg, Copenhagen, NV Denmark

**Keywords:** Outcomes research, Health care, Medical research, Osteoarthritis

## Abstract

“Fast-track” protocols has improved surgical care with a reduction in length of hospital stay (LOS) in total hip (THA) and knee arthroplasty (TKA). However, the effects of continuous refinement of perioperative care lack detailed assessment. We studied time-related changes in LOS and morbidity after THA and TKA within a collaboration with continuous scientific refinement of perioperative care. Prospective multicentre consecutive cohort study between 2010 and 2017 from nine high-volume orthopaedic centres with established fast-track THA and TKA protocols. Prospective collection of comorbidities and complete 90-day follow-up from the Danish National Patient Registry and medical records. Of 36,935 procedures median age was 69 [62 to 75] years and 58% women. LOS declined from three [two to three] days in 2010 to one [one to two] day in 2017. LOS > 4 days due to “medical” or “surgical” complications, and “with no recorded morbidity” declined from 4.4 to 2.7%, 1.5 to 0.6%, and 3.8 to 1.3%, respectively. 90-days readmission rate declined from 8.6 to 7.7%. Our multicentre study in a socialized healthcare setting was associated with a continuous reduction in LOS and morbidity after THA and TKA.

## Introduction

Total hip (THA) and knee (TKA) arthroplasty are common procedures and continuously increasing throughout the last decades^1–3^, as the population grows older and surgical and perioperative management are refined. However, although THA and TKA are fairly “safe” procedures, the expenses related to the vast number of procedures may strain healthcare economics. In the early 00’s, the introduction of “enhanced recovery after surgery” (ERAS) or “fast-track” protocols in THA and TKA drastically improved postoperative recovery^4^.

Several European “before” and “after” series of fast-track THA and TKA’s have reported a marked decrease in length of stay (LOS) from median five-six to three days, with reduced postoperative morbidity and no increased readmission rates^5–7^. Consequently, the advantages of a fast-track protocol providing the “best evidence based care” may be considerable, especially within a publicly financed healthcare system. Despite this apparent success, other large series have shown problems with implementation of most recent evidence-based care principles. In this context, a recent UK analysis in more than 400,000 TKA’s investigating the association of national ERAS implementation while adjusting for time trends in LOS found no benefits of ERAS implementation, but without details on the ERAS protocols^8^. . Also, between 2008 and 2016, mean LOS was only reduced by two days from mean 5.8 to 3.7 days^8^. This may be considered somewhat disappointing compared to other studies with mean/median LOS around of three days^5–7^. Additionally, a recent large Canadian regional database study on more than 200,000 TKAs performed between 2003 and 2016, reported a decrease in LOS from median five to three days^9^. From the US, reports from large series/databases have shown reductions in LOS from mean about four to three days^10,11^ and median from three to two days^3^, but these studies have several methodological limitations. Thus, there has often been a lack of important information on context^12^ e.g. organisational factors, the perioperative setting, and completeness and validity of data^13^. These factors are important in generalizability of the results but also in clinical implementation of the investigated perioperative setting^14^. In summary, despite ERAS protocols for THA and TKA have been available and successful for more than a decade, quality assurance data with continuous outcome-monitoring on LOS, complications and readmissions are limited, especially with use of contemporary evidence-based analgesic techniques, rehabilitation protocols, blood management and optimized organisational pathways.

In this context, the Lundbeck Foundation Centre for Fast-track Hip and Knee replacement (www.fthk.dk) was established in 2009 as a multicentre collaboration of Danish high-volume ERAS departments, based upon the public socialised health care system in Denmark, contributing to about 50% of the Danish THA/TKA production. This collaboration was based on the unique monitoring possibilities with several nationwide registries in Denmark and furthermore included one to two yearly meetings on outcome monitoring and scientific collaboration with more than 170 publications (www.fthk.dk). Consequently, we aimed at investigating time-related changes in LOS, cause of LOS > 4 days and 30- and 90-days complete readmission and mortality rates in unselected elective unilateral fast-track THA and TKA from this research-based multicentre collaboration with a well-established but continuously developing fast-track setup.

## Results

**Figure 1:**
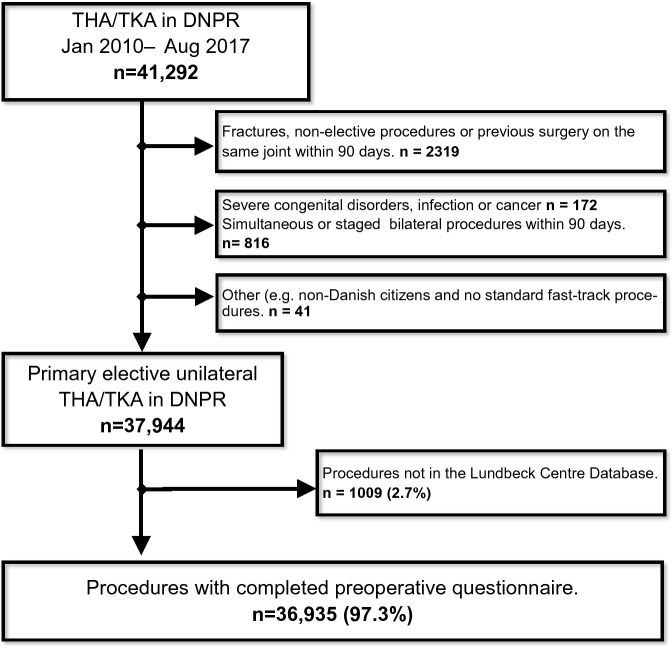
Flowchart of study population. *DNPR* Danish National Patient Registry, *THA* total hip arthroplasty, *TKA* total knee arthroplasty.

**Figure 2:**
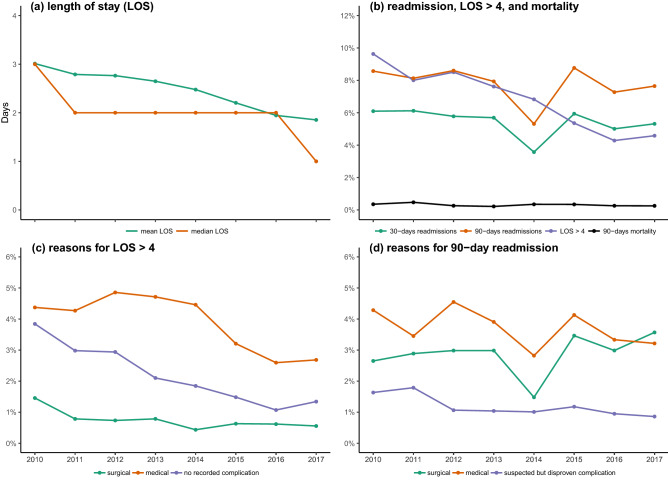
Temporal trends in length of stay (LOS), readmissions and mortality from 2010–2017 in 36,935 fast-track THA and TKAs. (**a**) Mean and median LOS, (**b**) proportion of procedures with LOS > 4 days, 90-day readmission and mortality rate, (**c**) proportion of procedures with “surgical”-, “medical”-. and no recorded morbidity as reason for LOS > 4 days, (**d**) readmissions due to “surgical”-, “medical”-, and disproven complications. Figure created using R (R: A Language and Environment for Statistical Computing, Version 3.6.1; 2019) and ggplot (ggplot2: Elegant Graphics for Data Analysis, version 3.3.0, 2016).

We included 36,935 procedures (19,977 (54.1%) THA) in 32,515 patients (Fig. 1) of which 21,524 (58.3%) were women and median age was 69 [Q1-Q3; 62–75] years. Median LOS was 2 [1 to 3] days with 2391 (6.5%) having a LOS > 4 days. LOS decreased throughout the study period from median three [2 to 3] days (mean 3.0 (± 2.4) days) in 2010 to one [1 to 2] day (mean 1.9 (± 1.6) days) in 2017 (Mann–Kendall p = 0.049) (Fig. 2a). Likewise, the proportion with LOS > four days decreased from 9.7% in 2010 to 4.6% in 2017 (Mann–Kendall p = 0.004) (Fig. 2b). The 30- and 90-day readmission rates decreased insignificantly from 6.1% and 8.6% in 2010 to 5.3% and 7.7% in 2017 (p = 0.107 and 0.386), respectively (Fig. 2b).

**Figure 3:**
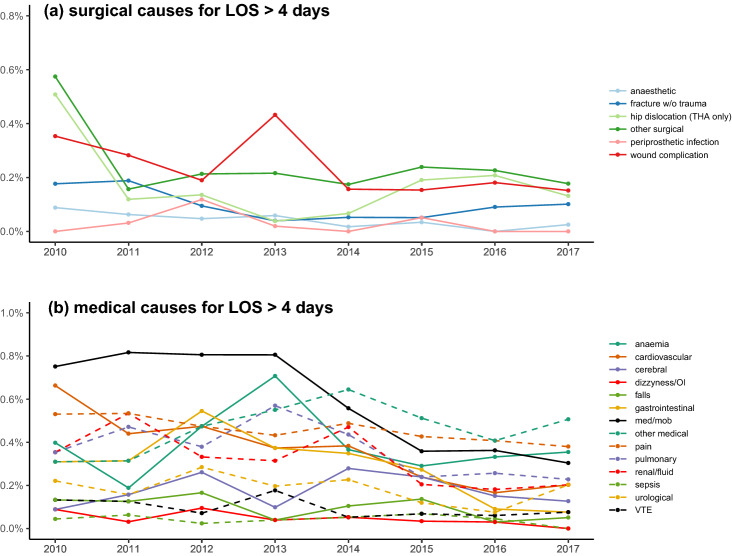
Temporal trends in specific causes for LOS > 4 days in 36,935 fast-track THA and TKAs. (**a**) “surgical” causes; (**b**) “medical” causes. Figure created using R (R: A Language and Environment for Statistical Computing, Version 3.6.1; 2019) and ggplot (ggplot2: Elegant Graphics for Data Analysis, version 3.3.0, 2016).

In-hospital “medical” complications leading to LOS > 4 days did not decline monotonically from 2010 to 2017 (Mann–Kendall p = 0.108), but with a constant decrease starting in 2014 (4.4% in 2014 to 2.7% in 2017). In contrast, the fraction of patients with LOS > 4 days due to “surgical” complications decreased continuously from 1.5% in 2010 to 0.6% in 2017 (Mann–Kendall p = 0.035). Also, the proportion of patients with no recorded postoperative morbidity but LOS > 4 days (3.8% in 2010 and 1.3% in 2017) was monotonically decreasing throughout the period (Mann–Kendall p = 0.002) (Fig. 2c). For specific causes of LOS > 4 days see Fig. 3 and Supplemental Table 1.

**Figure 4:**
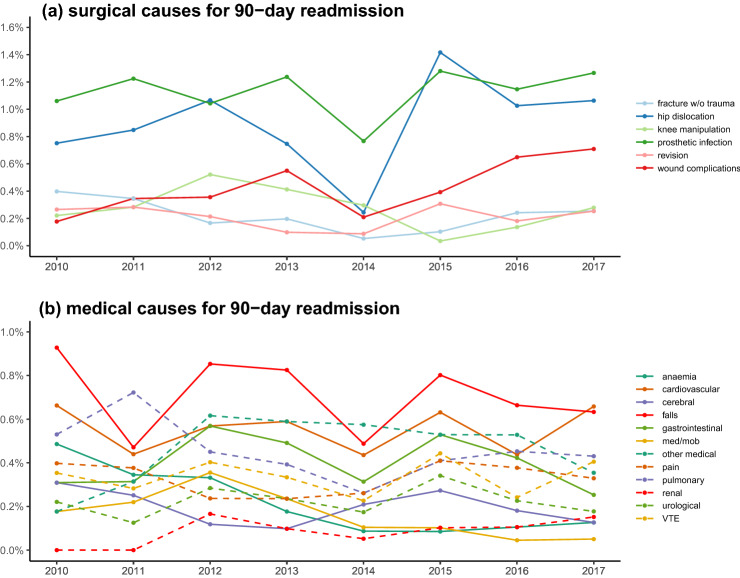
Temporal trends in specific causes for 90-day readmission in 36,935 fast-track THA and TKA. (**a**) “surgical” causes; (**b**) “medical” causes. Figure created using R (R: A Language and Environment for Statistical Computing, Version 3.6.1; 2019) and ggplot (ggplot2: Elegant Graphics for Data Analysis, version 3.3.0, 2016).

Regarding 90-day readmissions “surgical” complications increased slightly from 2.7% in 2010 to 3.6% in 2017 (p = 0.063), but with a pronounced yearly variation (Fig. 2d). “Medical” complications decreased insignificantly from 4.3% in 2010 to 3.2% in 2017 (p = 0.174) (Fig. 2d). Finally, the proportion with suspected but disproven morbidity (mostly thromboembolic complications) decreased continuously from 1.6% in 2010 to 0.9% in 2017 (p = 0.019) (Fig. 2d). See Fig. 4a,b and Supplemental Table 2 for specific causes of 90-day readmissions including multiple readmissions.

Overall, there were 112 (0.30%) all-cause deaths at 90 days, without changes throughout the study period (p = 0.108) (Fig. 2b). Of the 112 deaths, 72 were “surgically” related (0.19%) after median 18.5 [5.25–38.0] days and 27 (0.07%) were not related to surgery and occurring after median 59.5 [39.25–75.50] days. Finally, 13 (0.04%) patients died at home of unknown causes after median 68 [52–82.25] days (Supplemental Table 3). Thus, a worst-case scenario of “surgically” related 90-day mortality results in a rate of 0.23% when including deaths of unknown causes.

**Figure 5:**
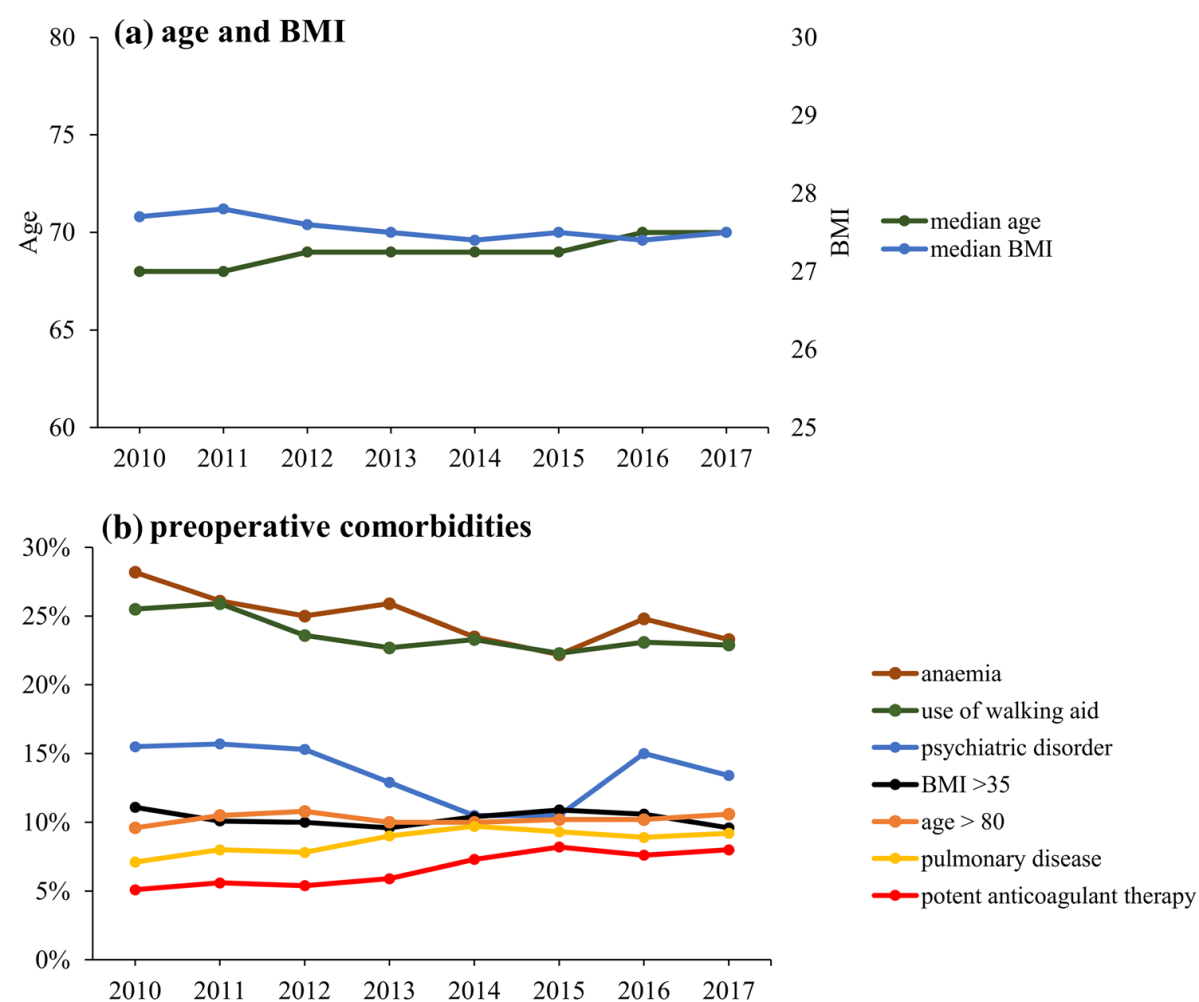
Temporal trends of preoperative characteristics of 36,935 fast-track THA and TKA procedures. (**a**) Median age and BMI; (**b**) preoperative comorbidities*;* potent anticoagulants: preoperative vitamin-k antagonists and direct oral anticoagulant. Figure created using R (R: A Language and Environment for Statistical Computing, Version 3.6.1; 2019) and ggplot (ggplot2: Elegant Graphics for Data Analysis, version 3.3.0, 2016).

Median age of patients increased from 68 [61–75] years in 2010 to 70 [62–76] years in 2017, without changes in BMI (Fig. 5a). The proportions of patients with age ≥ 80 years, BMI ≥ 35, use of walking aids, preoperative potent anticoagulant use, anaemia, psychiatric disorders and pulmonary disease were unchanged throughout the period (Fig. 5b). Furthermore, the proportion of patients with two or more of the above-mentioned characteristics remained stable around 22%.

## Discussion

For the first time we have documented detailed trends in LOS, readmissions and specific complications within a well-established prospective dedicated multicentre fast-track THA and TKA collaboration during a seven-year period. Our data demonstrated a continuous reduction in LOS from median three to one day (mean 3.0 to 1.9 days), with fewer patients with LOS > 4 days, and insignificant decreases in 30- and 90-day readmissions, but unchanged low “surgically” related mortality about 0.2%.

A reduction in LOS (median six to three days) without increase in readmission (4.7% vs 4.6%) after implementation of a fast-track protocol has been shown in 2014 on 3,000 fast-track procedures from the United Kingdom based on NHS data^5^. Likewise, a Swedish multicentre cohort study from 2011 to 2015 on 7270 fast-track procedures from national arthroplasty- and regional patient registers found decreasing LOS from median five to three days without changes in 90-day readmission rates after implementation of the fast-track protocol^7^. From the US, a large study using the Premiere Healthcare claims database demonstrated a reduced LOS of about 1 day ( from about 3 to 2 days) between 2006 and 2016 depending on degree of adherence to enhanced recovery components^15^. Also, in 2017 the NSQIP database demonstrated LOS ≤ 1 day in only about 30% of procedures^16^. However, these results are somewhat difficult to interpret due to limited information on use of post-discharge facilities and emergency/short-term readmissions. Recently a study on national implementation of ERAS in TKA in the UK using NHS data from 2008 to 2016 found a time-related reduction in LOS from mean 5.8 to 3.7 days but without additional benefits of the attempted national ERAS implementation^8^. However, patients with LOS > 15 days were excluded, and there was no clear description of the ERAS protocol, and no analysis of reasons for prolonged LOS or complications. However, considering that our participating departments already had a decade’s experience with fast-track protocols^17^, the continuous reduction in LOS from median three to one day and morbidity is encouraging. Thus, our results demonstrate that even years after implementation of fast-track protocols, a continued focus and refinement of well-established fast-track protocols with implementation of e.g. high dose methylprednisolone^18^, tranexamic acid, and no routine use of tourniquets^19^, drains and urinary catheters^20^ in combination may lead to further reduction in LOS.

Some of the reduction may be attributed to improved logistics regarding discharge, as suggested by the monotonic decrease in the proportion of patients with no mentioned morbidity but a LOS of > 4 days. Potential logistic improvement may also have been influenced by the introduction of outpatient THA and TKA surgery at two of the collaborating centres^21^. However, improved logistics is a key factor in our fast-track protocol and the individual components have undergone several scientific evaluations and discussions for implementation at the annual scientific meetings. Thus, we believe that improved logistics together with scientific documentation of the individual care component (www.fthk.dk) in combination may have contributed to the decreasing LOS.

Despite the absence of monotonic trends in LOS > 4 days due “medical” complications, there was a continuous drop in the number of these from 2014 and onwards. Interestingly, from 2014 the departments introduced standard high-dose preoperative methylprednisolone in TKA and increasingly also in THA based upon RCTs and the demonstrated improved analgesia, opioid-sparing, reduced nausea and vomiting without safety issues^18^.

A year to year reduction in most specific complications has been demonstrated previously using national UK registry data, but with increased occurrence of lower respiratory tract infection (0.54% to 0.84%) and renal failure (0.21% to 1.09%) from 2005 to 2014^22^. A recent study from the US using the Premier Database but without information on ERAS compliance, also reported a general decreasing complication rates, but increasing occurrence of renal insufficiency, potentially attributable to increasing comorbidity or changes in coding practice^3^. In contrast, we found no increase in renal or pulmonary complications within our data.

Early multidisciplinary instructive physiotherapy^23^ and nurse-led centred daily care and discharge is among the cornerstones of the early postoperative in-hospital rehabilitation^24^. Generally, the use and availability of discharge to skilled nursing facilities is rare in Denmark. Thus, a prior publication from the current cohort in 549 patients ≥ 85 years found that only 7% were discharged to other destinations than home and mainly from one institution^25^. In contrast, studies from other countries have reported discharge to other destinations than own home in 20–50% of patients^2,26^.

The minor decline in readmissions combined with the profound decline in in-hospital morbidity leading to LOS > 4 days suggest that the continuous effect on the fast-track protocol has primarily been on in-hospital complications. Importantly, there were only minor yearly fluctuations in post-discharge VTE rates which were consistently < 0.2%, confirming previous safety studies of “in-hospital” prophylaxis only in patients with LOS ≤ 5 days^27^. In contrast, there was a tendency towards increased “surgical” readmissions from 2.7% in 2010 to 3.6% in 2017 as demonstrated by the lack of improvements in hip dislocations and wound complications (Fig. 4a, Supplemental Table 2).

We found no changes in 90-day mortality, neither when looking at all-cause or “surgically “related mortality^28^, while other large national registry studies have found decreasing mortality, although not specifically related to a fast-track setup and with limitations in follow-up completeness and time, limiting comparisons^3,5,22,29^.

Although we were able to achieve highly detailed information through the combination of complete follow-up from a national registry, chart review, and prospective collection of preoperative risk-factors there are some limitations. Thus, the observational design without a control group eliminates the possibility to compare with “conventional” care. However, this was not the aim of our study as we intended to report time trends in perioperative outcomes within a clinical and scientific collaboration dedicated to further improvements of perioperative care. For such evaluations an observational design may be of considerable value^30^. Also, we only included in-hospital complications resulting in LOS > 4 days in the analysis, potentially missing minor complications within the first four days. The inclusion of patients with multiple procedures within the study period, could also potentially bias our results^31^, but the same may happen if leaving them out^32^. Finally, 2.7% of the performed procedures were not registered in the LCDB and consequently excluded from the study. However, a previous investigation on the excluded patients did not find any difference in outcome for these patients vs those in LCDB^33^. Thus, we believe the continuous improvements in our “real-life” multicentre cohort of unselected elective primary THA and TKA are generalizable and hopefully may stimulate to continued implementation with adherence to contemporary evidence-based fast-track protocols with regular audit of own data. However, we acknowledge that this large detailed cohort does not provide patient reported outcomes or surgeon specific data, since we aimed to assess whether large-scale improvements could be obtained from the entire care pathway and scientific collaboration rather than individual interventions.

In conclusion, our scientific multicentre eight-year collaboration in a socialized healthcare setting was associated to a continuous reduction in LOS to median one day and lower morbidity after THA and TKA. A continuous use and refinement of science-based fast-track protocols may lead to further reduction in LOS and morbidity^23^.

## Methods

We used an observational study design on a prospectively collected consecutive cohort of unselected primary elective unilateral THA and TKA’s from January 2010 to August 2017. The procedures were performed at nine dedicated fast-track centres reporting to the Lundbeck Foundation Centre for Fast-track Hip and Knee Replacement database (LCDB)^34^ and accounting for around 50% of the annual Danish TKA and THA’s. The LCDB originally consisted of six orthopaedic centres with an additional three centres joining in 2012, 2013, and 2014, respectively. All centres used fast-track protocols focusing on neuraxial anaesthesia, multimodal opioid sparing analgesia, in-hospital only thromboprophylaxis if LOS ≤ 5 days, early (< 6 h postoperatively) mobilization, and discharge to own home based on functional discharge criteria^20^. Minor protocol changes were implemented continuously during the collaboration based upon the study results (www.fthk.dk) e.g. perioperative high-dose glucocorticoid since 2014^18^, avoidance of tourniquet since 2014^19^, and reduced bladder catherization since 2015^35,36^. The reporting of the study follows the “Strengthening the Reporting of Observational studies in Epidemiology” (STROBE statement)^37^ and the “Standards for QUality Improvement Reporting Excellence” (SQUIRE 2.0) guideline^38^. Following Danish law approval from the ethics committee was not required due to the non-interventional nature of the study. The Danish National Board of Health (3-3013-56/2/EMJO) and the Danish Data Protection Agency (RH-2017-132) gave permission to review and store patient records without informed consent. The LCDB is registered as an ongoing study registry on ClinicalTrials.gov (NCT01515670). Thus, the study was conducted in accordance with relevant guidelines and regulations.

Data on demographics and preoperative comorbidity were prospectively collected by a nurse-assisted patient reported questionnaire entered into the LCDB^34^. Data on LOS, 90-day readmission and mortality were obtained from the Danish National Patient Registry (DNPR) to which reporting is mandatory for all hospitals in Denmark securing complete (> 99%) follow-up^39^. LOS was calculated as postoperative nights in hospital including transfers to other departments and hospitals. Readmissions were defined as unplanned admissions with ≥ 1 night in hospital and potentially related to the index surgical procedure. In case of LOS > 4 days, 90-day readmission or mortality, discharge summaries were obtained from the departments and scrutinized for reason for prolonged hospitalization, cause of readmission and/or mortality. In case of doubt complete health care records were obtained. Readmissions without possible relation to index procedure were excluded (e.g. eye surgery, unrelated cancer surgery, planned follow-up on unrelated conditions and other obviously unrelated readmissions). Investigation of discharge summaries and health records were done by CJ from Jan 2010 to Sep 2013, and from Oct 2013 to Aug 2017 by PBP supervised by CJ. In case of doubt the discharge summary and health records were discussed with all authors to obtain agreement on cause and possible relation to index procedure. Based on previous work we categorised causes of prolonged admission (LOS > 4 days) and readmission into “surgical” and “medical” complications (Table 1), both requiring potential relation to index procedure^33,34^. Additionally, we investigated potential changes in the proportion of patients with age ≥ 80 years, BMI ≥ 35, use of walking aids, preoperative potent anticoagulant use, anaemia, psychiatric disorders and pulmonary disease based on a previous investigations finding these variables being associated with “medical” complications^34^.

**Table 1:** Classification of “medical” versus “surgical” complications.

“Medical” complications	“Surgical” complications
Sepsis	Anaesthetic complication
Cardiac	Prosthetic infection
Gastrointestinal	Hip dislocation
Anaemia	Fractures without trauma
Venous thromboembolism	Wound complications
Medication/mobilization	Revision surgery
Fall	Knee manipulation under anaesthesia
Orthostatic intolerance/dizziness	
Urological	
Renal	
Pulmonary	
Cerebral	
Pain	
Other	

Inclusion criteria were primary unilateral arthroplasty in patients aged ≥ 18 years and with a Danish social security number. We excluded non-elective procedures, procedures due to fractures ≤ 90 days, and previous THA/TKA ≤ 90 days.

The majority of the current cohort (36,608 procedures) was included in a preliminary study superficially describing developments in LOS and 90-day readmissions during the same study period^40^. However, that study did not explore developments and role of specific complications leading to either prolonged LOS or readmissions, nor did it investigate mortality or changes in patient characteristics.

### Objectives

The primary objective was to investigate changes in LOS and in-hospital morbidity resulting in LOS > 4 days, and 90-day readmissions over time. Secondary outcomes were analyses of specific in-hospital complications leading to LOS > 4 days and/or readmission ≤ 90 days and a division into composite outcomes of “surgical” and “medical” complications. Finally, all cause and “surgically” related 90-days mortality, 30-days readmission rate and changes in preoperative patient characteristics were investigated.

### Statistics

All eligible procedures within the study period were included and no pre-study power calculation was performed. Continuous data are presented as mean (SD) or median [IQR] and compared using Student’s t-test or Mann–Whitney *U* test as appropriate. Normality was assed using histograms and q–q plots. Categorical data are reported as actual number (%) and compared using chi-square or Fisher’s exact test as appropriate. When reporting readmission rates, “medical” and “surgical” morbidity rates we included only the first readmission. When reporting specific types of morbidity all readmissions were included. To investigate potential monotonic trends in proportions over time we used the Mann–Kendall test. We considered p < 0.05 as statistically significant. Initial data analysis was done using SPSS v.25 (IBM Corp, Armonk, NY, USA) while Mann–Kendall Analysis and plots were made in R (R Core Team, 2019) using the “Kendall” (Mcleod, 2011) and “ggplot2” (Wickham, 2016) packages.

## Supplementary information

Supplementary Tables.

## Data Availability

Limited data (deidentified preoperative questionnaire on comorbidities) is available for sharing upon reasonable request after approval from the Danish Data Protection Agency, due to data retrieval of the majority of variables from third parties.
