# Supplementary material for: Improvement in fast-track hip and knee arthroplasty: a prospective multicentre study of 36,935 procedures from 2010 to 2017
Source: Sci Rep. 2020 Dec 4;10:21233. doi: 10.1038/s41598-020-77127-6 (PMC7718264; doi:10.1038/s41598-020-77127-6)
Supplement: Supplementary file 1 — Supplementary Tables. [file 41598_2020_77127_MOESM1_ESM.docx]

Improvement in fast-track hip and knee arthroplasty – a prospective multicentre study of 36,935 procedures from 2010-2017

Pelle Baggesgaard Petersen^1,*^, Henrik Kehlet^1,2^, Christoffer Calov Jørgensen^1,2,*^; on behalf of the Lundbeck Foundation Centre for Fast-track Hip and Knee Replacement Collaborative Group.

Pelle B. Petersen and Christoffer C. Jørgensen contributed equally to this paper.

^1^Section for Surgical Pathophysiology, Rigshospitalet, Copenhagen, Denmark; ^2^Lundbeck Foundation Centre for Fast-track Hip and Knee Arthroplasty, Copenhagen, Denmark.

Correspondence to PB Petersen: [Pelle.Petersen.01@regionh.dk](mailto:Pelle.Petersen.01@regionh.dk)

Section for Surgical Pathophysiology, 7621

Copenhagen University Hospital, Rigshospitalet

Blegdamsvej 9

DK-2100 Copenhagen

Denmark

Phone: +4526792073

Running headline: Fast-track hip and knee arthroplasty

# **Supplementary files**

| **Supplemental table 1: Reasons for LOS > 4 days in 36935 fast-track THA/TKA patients** | | | | | | | | | | | | | | | | | | |
| --- | --- | --- | --- | --- | --- | --- | --- | --- | --- | --- | --- | --- | --- | --- | --- | --- | --- | --- |
|  | 2010 | | 2011 | | 2012 | | 2013 | | 2014 | | 2015 | | 2016 | | | 20171 | | |
|  | n (%) | | n (%) | | n (%) | | n (%) | | n (%) | | n (%) | | n (%) | | | n (%) | | |
| **Procedures** | 2263 | | 3185 | | 4220 | | 5091 | | 5740 | | 5860 | | 6627 | | | 3949 | | |
| **"Surgical" morbidity** | 33 | (1.5%) | 25 | (0.8%) | 31 | (0.7%) | 40 | (0.8%) | 25 | (0.4%) | 37 | (0.6%) | 41 | (0.6%) | 21 | | (0.5%) |  |
| anaesthetic complication | 2 | (0.1%) | 2 | (0.1%) | 2 | (0.0%) | 3 | (0.1%) | 1 | (0.0%) | 2 | (0.0%) | 0 | (0.0%) | 1 | | (0.0%) |  |
| Oozing/ haematoma | 8 | (0.4%) | 9 | (0.3%) | 8 | (0.2%) | 22 | (0.4%) | 9 | (0.2%) | 9 | (0.2%) | 12 | (0.2%) | 6 | | (0.2%) |  |
| Fracture without trauma | 4 | (0.2%) | 6 | (0.2%) | 4 | (0.1%) | 2 | (0.0%) | 3 | (0.1%) | 3 | (0.1%) | 6 | (0.1%) | 4 | | (0.1%) |  |
| Hip dislocation^2^ | 6 | (0.5%) | 2 | (0.1%) | 3 | (0.1%) | 1 | (0.0%) | 2 | (0.1%) | 6 | (0.2%) | 8 | (0.2%) | 3 | | (0.1%) |  |
| Periprosthetic infections | 0 | (0.0%) | 1 | (0.0%) | 5 | (0.1%) | 1 | (0.0%) | 0 | (0.0%) | 3 | (0.1%) | 0 | (0.0%) | 0 | | (0.0%) |  |
| Other surgical | 13 | (0.6%) | 5 | (0.2%) | 9 | (0.2%) | 11 | (0.2%) | 10 | (0.2%) | 14 | (0.2%) | 15 | (0.2%) | 7 | | (0.2%) |  |
| **"Medical" morbidity** | 99 | (4.4%) | 136 | (4.3%) | 205 | (4.9%) | 240 | (4.7%) | 256 | (4.5%) | 188 | (3.2%) | 172 | (2.6%) | 107 | | (2.7%) |  |
| sepsis | 1 | (0.0%) | 2 | (0.1%) | 1 | (0.0%) | 2 | (0.0%) | 3 | (0.1%) | 4 | (0.1%) | 3 | (0.0%) | 0 | | (0.0%) |  |
| cardiac | 15 | (0.7%) | 14 | (0.4%) | 20 | (0.5%) | 19 | (0.4%) | 22 | (0.4%) | 14 | (0.2%) | 11 | (0.2%) | 8 | | (0.2%) |  |
| gastrointestinal | 7 | (0.3%) | 10 | (0.3%) | 23 | (0.5%) | 19 | (0.4%) | 20 | (0.3%) | 16 | (0.3%) | 6 | (0.1%) | 3 | | (0.1%) |  |
| anaemia | 9 | (0.4%) | 6 | (0.2%) | 20 | (0.5%) | 36 | (0.7%) | 21 | (0.4%) | 17 | (0.3%) | 22 | (0.3%) | 14 | | (0.4%) |  |
| Venous thromboembolism | 3 | (0.1%) | 4 | (0.1%) | 3 | (0.1%) | 9 | (0.2%) | 3 | (0.1%) | 4 | (0.1%) | 4 | (0.1%) | 3 | | (0.1%) |  |
| Medication/Mobilisation | 17 | (0.8%) | 26 | (0.8%) | 34 | (0.8%) | 41 | (0.8%) | 32 | (0.6%) | 21 | (0.4%) | 24 | (0.4%) | 12 | | (0.3%) |  |
| fall | 3 | (0.1%) | 4 | (0.1%) | 7 | (0.2%) | 2 | (0.0%) | 6 | (0.1%) | 8 | (0.1%) | 2 | (0.0%) | 2 | | (0.1%) |  |
| orthostatic intolerance / dizziness | 2 | (0.1%) | 1 | (0.0%) | 4 | (0.1%) | 2 | (0.0%) | 3 | (0.1%) | 2 | (0.0%) | 2 | (0.0%) | 0 | | (0.0%) |  |
| renal/fluid related | 8 | (0.4%) | 17 | (0.5%) | 14 | (0.3%) | 16 | (0.3%) | 27 | (0.5%) | 12 | (0.2%) | 12 | (0.2%) | 8 | | (0.2%) |  |
| urological | 5 | (0.2%) | 5 | (0.2%) | 12 | (0.3%) | 10 | (0.2%) | 13 | (0.2%) | 7 | (0.1%) | 5 | (0.1%) | 8 | | (0.2%) |  |
| pulmonary | 8 | (0.4%) | 15 | (0.5%) | 16 | (0.4%) | 29 | (0.6%) | 25 | (0.4%) | 14 | (0.2%) | 17 | (0.3%) | 9 | | (0.2%) |  |
| cerebral | 2 | (0.1%) | 5 | (0.2%) | 11 | (0.3%) | 5 | (0.1%) | 16 | (0.3%) | 14 | (0.2%) | 10 | (0.2%) | 5 | | (0.1%) |  |
| pain | 12 | (0.5%) | 17 | (0.5%) | 20 | (0.5%) | 22 | (0.4%) | 28 | (0.5%) | 25 | (0.4%) | 27 | (0.4%) | 15 | | (0.4%) |  |
| other medical | 7 | (0.3%) | 10 | (0.3%) | 20 | (0.5%) | 28 | (0.5%) | 37 | (0.6%) | 30 | (0.5%) | 27 | (0.4%) | 20 | | (0.5%) |  |
| **No recorded morbidity** | 87 | (3.8%) | 95 | (3.0%) | 124 | (2.9%) | 107 | (2.1%) | 106 | (1.8%) | 87 | (1.5%) | 71 | (1.1%) | 53 | | (1.3%) |  |
| **LOS > 4 days** | 219 | (9.7%) | 256 | (8.0%) | 360 | (8.5%) | 387 | (7.6%) | 387 | (6.7%) | 312 | (5.3%) | 284 | (4.3%) | 181 | | (4.6%) |  |
| ^1^Until August 2017; ^2^ Of total hip arthroplasties | | | | | | | | | | | | | | | | | | |

| **Supplemental table 2: Reasons for 90-days readmission after 36,935 fast-track THA/TKA.** | | | | | | | | | | | | | | | | |
| --- | --- | --- | --- | --- | --- | --- | --- | --- | --- | --- | --- | --- | --- | --- | --- | --- |
|  | 2010 | | 2011 | | 2012 | | 2013 | | 2014 | | 2015 | | 2016 | | 2017 | |
|  | n (%) | | n (%) | | n (%) | | n (%) | | n (%) | | n (%) | | n (%) | | n (%) | |
| **Procedures** | 2263 | | 3185 | | 4220 | | 5091 | | 5740 | | 5860 | | 6627 | | 3949 | |
| **"Surgical" morbidity** | 65 | (2.9%) | 106 | (3.3%) | 142 | (3.4%) | 165 | (3.2%) | 95 | (1.7%) | 207 | (3.5%) | 224 | (3.4%) | 151 | (3.8%) |
| Prosthetic infections | 24 | (1.1%) | 39 | (1.2%) | 44 | (1.0%) | 63 | (1.2%) | 44 | (0.8%) | 75 | (1.3%) | 76 | (1.1%) | 50 | (1.3%) |
| Wound complications | 4 | (0.2%) | 11 | (0.3%) | 15 | (0.4%) | 28 | (0.5%) | 12 | (0.2%) | 23 | (0.4%) | 43 | (0.6%) | 28 | (0.7%) |
| Fracture without trauma | 9 | (0.4%) | 11 | (0.3%) | 7 | (0.2%) | 10 | (0.2%) | 3 | (0.1%) | 6 | (0.1%) | 16 | (0.2%) | 10 | (0.3%) |
| Hip dislocation^1^ | 17 | (1.4%) | 27 | (1.6%) | 45 | (2.0%) | 38 | (1.4%) | 14 | (0.5%) | 83 | (2.6%) | 68 | (1.8%) | 42 | (1.8%) |
| Revision | 6 | (0.3%) | 9 | (0.3%) | 9 | (0.2%) | 5 | (0.1%) | 5 | (0.1%) | 18 | (0.3%) | 12 | (0.2%) | 10 | (0.3%) |
| Knee manipulation^2^ | 5 | (0.5%) | 9 | (0.6%) | 22 | (1.1%) | 21 | (0.9%) | 17 | (0.6%) | 2 | (0.1%) | 9 | (0.3%) | 11 | (0.7%) |
| **"Medical" morbidity** | 98 | (4.3%) | 119 | (3.7%) | 204 | (4.8%) | 212 | (4.2%) | 175 | (3.0%) | 269 | (4.6%) | 249 | (3.8%) | 146 | (3.7%) |
| Cardiac | 10 | (0.4%) | 10 | (0.3%) | 19 | (0.5%) | 23 | (0.5%) | 17 | (0.3%) | 33 | (0.6%) | 27 | (0.4%) | 26 | (0.7%) |
| Gastrointestinal | 7 | (0.3%) | 10 | (0.3%) | 24 | (0.6%) | 25 | (0.5%) | 18 | (0.3%) | 31 | (0.5%) | 28 | (0.4%) | 10 | (0.3%) |
| Anaemia | 11 | (0.5%) | 11 | (0.3%) | 14 | (0.3%) | 9 | (0.2%) | 5 | (0.1%) | 5 | (0.1%) | 7 | (0.1%) | 5 | (0.1%) |
| Venous thromboembolism | 8 | (0.4%) | 9 | (0.3%) | 17 | (0.4%) | 17 | (0.3%) | 13 | (0.2%) | 26 | (0.4%) | 16 | (0.2%) | 16 | (0.4%) |
| Medication/Mobilization | 4 | (0.2%) | 7 | (0.2%) | 15 | (0.4%) | 12 | (0.2%) | 6 | (0.1%) | 6 | (0.1%) | 3 | (0.0%) | 2 | (0.1%) |
| Falls | 21 | (0.9%) | 15 | (0.5%) | 36 | (0.9%) | 42 | (0.8%) | 28 | (0.5%) | 47 | (0.8%) | 44 | (0.7%) | 25 | (0.6%) |
| Urological | 5 | (0.2%) | 4 | (0.1%) | 12 | (0.3%) | 12 | (0.2%) | 10 | (0.2%) | 20 | (0.3%) | 15 | (0.2%) | 7 | (0.2%) |
| Pulmonary | 12 | (0.5%) | 23 | (0.7%) | 19 | (0.5%) | 20 | (0.4%) | 15 | (0.3%) | 24 | (0.4%) | 30 | (0.5%) | 17 | (0.4%) |
| Cerebral | 7 | (0.3%) | 8 | (0.3%) | 5 | (0.1%) | 5 | (0.1%) | 12 | (0.2%) | 16 | (0.3%) | 12 | (0.2%) | 5 | (0.1%) |
| Pain | 9 | (0.4%) | 12 | (0.4%) | 10 | (0.2%) | 12 | (0.2%) | 15 | (0.3%) | 24 | (0.4%) | 25 | (0.4%) | 13 | (0.3%) |
| Renal | 0 | (0.0%) | 0 | (0.0%) | 7 | (0.2%) | 5 | (0.1%) | 3 | (0.1%) | 6 | (0.1%) | 7 | (0.1%) | 6 | (0.2%) |
| Other | 4 | (0.2%) | 10 | (0.3%) | 26 | (0.6%) | 30 | (0.6%) | 33 | (0.6%) | 31 | (0.5%) | 35 | (0.5%) | 14 | (0.4%) |
| **Suspected but disproven complication** | 45 | (2.0%) | 66 | (2.1%) | 50 | (1.2%) | 63 | (1.2%) | 67 | (1.2%) | 79 | (1.3%) | 65 | (1.0%) | 40 | (1.0%) |
| DVT/ PE | 29 | (1.3%) | 43 | (1.4%) | 27 | (0.6%) | 37 | (0.7%) | 43 | (0.7%) | 56 | (1.0%) | 52 | (0.8%) | 33 | (0.8%) |
| Infection | 11 | (0.5%) | 19 | (0.6%) | 18 | (0.4%) | 19 | (0.4%) | 16 | (0.3%) | 19 | (0.3%) | 11 | (0.2%) | 7 | (0.2%) |
| Myocardial Infarction | 5 | (0.2%) | 4 | (0.1%) | 5 | (0.1%) | 7 | (0.1%) | 8 | (0.1%) | 4 | (0.1%) | 2 | (0.0%) | 0 | (0.0%) |
| **90-days readmission rate** | 208 | (9.2%) | 291 | (9.1%) | 396 | (9.4%) | 440 | (8.6%) | 337 | (5.9%) | 555 | (9.5%) | 538 | (8.1%) | 337 | (8.5%) |
| Numbers differ from reported rates due to inclusion of multiple readmissions in specific cause of 90-day readmissions. ^1^Of total hip arthroplasties; ^2^ of total knee arthroplasties. | | | | | | | | | | | | | | | | |

| **Supplemental table 3: Causes of 90-days mortality after 36,935 fast-track THA/TKA's** | | | | | | | | | | | | | | | | | | |
| --- | --- | --- | --- | --- | --- | --- | --- | --- | --- | --- | --- | --- | --- | --- | --- | --- | --- | --- |
|  | 2010 | | 2011 | | 2012 | | 2013 | | 2014 | | 2015 | | 2016 | | 2017 | | Total | |
|  | n (%) | | n (%) | | n (%) | | n (%) | | n (%) | | n (%) | | n (%) | | n (%) | | n (%) | |
| Procedures | 2263 | | 3185 | | 4220 | | 5091 | | 5740 | | 5860 | | 6627 | | 3949 | | 36935 | |
| Cardiac | 1 | (0.04%) | 1 | (0.03%) | 1 | (0.02%) | 1 | (0.02%) | 5 | (0.09%) | 3 | (0.05%) | 1 | (0.02%) | 4 | (0.10%) | 17 | (0.05%) |
| Pulmonary embolism | 0 | (0.00%) | 2 | (0.06%) | 2 | (0.05%) | 0 | (0.00%) | 3 | (0.05%) | 1 | (0.02%) | 1 | (0.02%) | 0 | (0.00%) | 9 | (0.02%) |
| Gastrointestinal | 1 | (0.04%) | 3 | (0.09%) | 3 | (0.07%) | 1 | (0.02%) | 2 | (0.03%) | 0 | (0.00%) | 0 | (0.00%) | 0 | (0.00%) | 10 | (0.03%) |
| Stroke | 1 | (0.04%) | 2 | (0.06%) | 2 | (0.05%) | 0 | (0.00%) | 2 | (0.03%) | 3 | (0.05%) | 2 | (0.03%) | 0 | (0.00%) | 12 | (0.03%) |
| Pulmonary | 2 | (0.09%) | 2 | (0.06%) | 1 | (0.02%) | 2 | (0.04%) | 0 | (0.00%) | 0 | (0.00%) | 3 | (0.05%) | 1 | (0.03%) | 11 | (0.03%) |
| Cancer | 1 | (0.04%) | 2 | (0.06%) | 2 | (0.05%) | 0 | (0.00%) | 0 | (0.00%) | 3 | (0.05%) | 1 | (0.02%) | 1 | (0.03%) | 10 | (0.03%) |
| Surgical comp | 0 | (0.00%) | 0 | (0.00%) | 0 | (0.00%) | 2 | (0.04%) | 1 | (0.02%) | 1 | (0.02%) | 0 | (0.00%) | 2 | (0.05%) | 6 | (0.02%) |
| Renal | 0 | (0.00%) | 1 | (0.03%) | 0 | (0.00%) | 0 | (0.00%) | 0 | (0.00%) | 1 | (0.02%) | 0 | (0.00%) | 0 | (0.00%) | 2 | (0.01%) |
| Unknown | 2 | (0.09%) | 2 | (0.06%) | 0 | (0.00%) | 5 | (0.10%) | 6 | (0.10%) | 8 | (0.14%) | 9 | (0.14%) | 2 | (0.05%) | 34 | (0.09%) |
| Other | 0 | (0.00%) | 0 | (0.00%) | 0 | (0.00%) | 0 | (0.00%) | 1 | (0.02%) | 0 | (0.00%) | 0 | (0.00%) | 0 | (0.00%) | 1 | (0.00%) |
| 90-days mortality | 8 | (0.35%) | 15 | (0.47%) | 11 | (0.26%) | 11 | (0.22%) | 20 | (0.35%) | 20 | (0.34%) | 17 | (0.26%) | 10 | (0.25%) | 112 | (0.30%) |
| Surgically related | 6 | (0.27%) | 10 | (0.31%) | 9 | (0.21%) | 6 | (0.12%) | 13 | (0.23%) | 13 | (0.22%) | 7 | (0.11%) | 8 | (0.20%) | 72 | (0.19%) |
| Not surgically related | 2 | (0.09%) | 5 | (0.16%) | 2 | (0.05%) | 5 | (0.10%) | 4 | (0.07%) | 5 | (0.09%) | 3 | (0.05%) | 1 | (0.03%) | 27 | (0.07%) |
| Unknown at own home | 0 | (0.00%) | 0 | (0.00%) | 0 | (0.00%) | 0 | (0.00%) | 3 | (0.05%) | 2 | (0.03%) | 7 | (0.11%) | 1 | (0.03%) | 13 | (0.04%) |
